# Differences in Micronutrient Knowledge, Beliefs, and Supplementation Practices Between Pregnant Women and Healthcare Providers: A Cross-Sectional Study

**DOI:** 10.3390/nu18121934

**Published:** 2026-06-15

**Authors:** Anna Elisabeth Hentrich, Dörthe Brüggmann, Samira Catharina Hoock, Lukas Jennewein, Frank Louwen, Eileen Deuster

**Affiliations:** Department of Obstetrics and Perinatal Medicine, University Hospital Frankfurt am Main, Goethe University Frankfurt, 60596 Frankfurt, Germany

**Keywords:** micronutrients, pregnancy, dietary supplements, nutritional knowledge, health communication, patient–provider differences, prenatal care, nutrition counseling

## Abstract

Background/Objectives: Adequate micronutrient intake during pregnancy is critical for fetal development, yet whether pregnant women and healthcare professionals share consistent knowledge, beliefs, and supplementation practices remains poorly characterized. Methods: Two parallel cross-sectional surveys using identical core items were conducted at a German tertiary care center between April and November 2024. Pregnant women (*n* = 132) and healthcare professionals who initiated the survey (*n* = 105) completed anonymous QR-code-based questionnaires assessing micronutrient-related knowledge, perceived dietary adequacy, and supplementation practices or recommendation patterns. Comparative analyses were restricted to fully completed healthcare professional questionnaires (*n* = 80). Group differences were analyzed using chi-square or Fisher’s exact tests. Results: Healthcare professionals demonstrated higher knowledge levels across most micronutrients. Knowledge gaps were most pronounced for vitamin B12, with 53.0% of pregnant women unable to identify any fetal effect compared with 20.0% of providers (*p* < 0.001). Beliefs about dietary sufficiency were broadly aligned for folic acid (*p* = 0.452) and vitamin D (*p* > 0.999), but diverged markedly for vitamin B12, where 79.2% of providers considered dietary intake alone adequate compared with 47.3% of pregnant women (*p* < 0.001). Substantial differences were observed between patient-reported supplementation practices and provider-reported recommendation patterns: Vitamin B12 (70.0% vs. 3.8%), vitamin D (76.2% vs. 41.3%), omega-3 fatty acids (76.2% vs. 47.5%), and folic acid (98.5% vs. 81.3%; all *p* < 0.001). The internet was the most frequently cited information source among pregnant women (89.4%), while healthcare professionals reported using both scientific literature (75.0%) and internet-based resources (76.3%), the latter primarily for accessing professional and scientific information. Conclusions: Substantial patient–provider differences in micronutrient knowledge, beliefs, and supplementation practices persist even within a highly educated population at a tertiary care center. These findings suggest potential differences between patient-reported supplementation behavior and provider-reported recommendation practices, particularly for vitamin B12 and vitamin D. These findings suggest that more structured communication regarding micronutrient supplementation during pregnancy is needed.

## 1. Introduction

Adequate micronutrient intake during pregnancy is essential for maternal health and optimal fetal development. Deficiencies in key nutrients such as folic acid, vitamin D, omega-3 fatty acids, and vitamin B12 have been associated with adverse outcomes, including neural tube defects, impaired skeletal mineralization, neurodevelopmental delay, and hematological disorders [1,2,3,4,5]. Reference intake recommendations during pregnancy include approximately 550 µg/day folate equivalents, 600–800 IU vitamin D, 3.5 µg/day vitamin B12, and at least 200 mg/day docosahexaenoic acid (DHA), although exact recommendations differ internationally and according to individual risk profiles [6,7,8]. The International Federation of Gynecology and Obstetrics (FIGO) emphasizes systematic assessment of maternal diet and micronutrient status and supports targeted supplementation with folic acid, iron, iodine, vitamin D, and long-chain omega-3 fatty acids, tailored to individual risk [9]. In Germany, national guidelines from the ‘Deutsche Gesellschaft für Ernährung (DGE)’ and the Federal Institute for Risk Assessment (BfR) recommend supplementation with folic acid from the periconceptional period, as well as iodine and, increasingly, vitamin D during pregnancy. While omega-3 fatty acid supplementation, particularly docosahexaenoic acid (DHA), is endorsed by professional bodies in light of its role in fetal brain and retinal development, formal inclusion in national guidelines remains inconsistent [2,10,11]. Vitamin B12 supplementation is specifically recommended for women following vegetarian or vegan diets, a dietary pattern of growing prevalence among women of childbearing age [5].

The translation of these recommendations into clinical practice depends not only on their availability but also on how effectively they are communicated and understood during antenatal care [12]. Healthcare professionals play a central role in providing nutritional counseling; however, previous studies indicate variability in provider knowledge and counselling practices, particularly for micronutrients beyond folic acid [13].

At the same time, pregnant women increasingly obtain health information from digital and non-clinical sources, which may influence their health-related decisions [14,15,16,17]. This creates the potential for misalignment between patients and providers across multiple domains, including knowledge of micronutrient function, beliefs regarding dietary adequacy, and supplementation behavior. Such differences may reflect variation in micronutrient-related knowledge, perceptions, and supplementation-related behavior between pregnant women and healthcare professionals.

To date, to our knowledge, no study has directly compared patients and providers using identical instruments within the same clinical setting. Such comparisons are particularly relevant in highly educated populations, where knowledge gaps might be expected to be minimal. Understanding whether alignment exists under these conditions may help identify structural gaps in nutritional communication and implementation in prenatal care.

The aim of this study was therefore to assess and compare micronutrient-related knowledge, perceived dietary adequacy for key nutrients, and supplementation practices among pregnant women and healthcare professionals at a German tertiary care center. By examining concordance and differences across these groups, this study seeks to identify potential gaps in micronutrient counseling and in the implementation of nutrient recommendations in prenatal care.

## 2. Materials and Methods

### 2.1. Study Design and Setting

This study employed a cross-sectional design using two parallel questionnaires administered to pregnant women and healthcare professionals (defined as practicing midwives, obstetricians, obstetric residents, or medical students working in obstetric practice) at the Department of Obstetrics and Prenatal Medicine, University Hospital Frankfurt, Germany, between April and November 2024.

The study was designed to enable direct comparison between these groups by incorporating an identical core set of items that assess knowledge and beliefs about key micronutrients during pregnancy. This study was designed as an exploratory cross-sectional analysis; therefore, no formal a priori sample size calculation was performed. The findings should be interpreted as hypothesis-generating.

Inclusion criteria were that the respondent should be over 18 years of age, be able to read and write German, and be either currently pregnant or working as a midwife, obstetrician, obstetric resident, or medical student at the study site.

### 2.2. Questionnaire Development

The two questionnaires were developed on the SoSci Survey platform (SoSci Survey GmbH, Munich, Germany), informed by international and German guidelines on micronutrient supplementation in pregnancy and previous survey instruments in this field [18,19]. Draft items were reviewed by a multidisciplinary team (obstetrician, midwife) for content relevance and wording. The questionnaires were then pilot-tested for clarity and face validity in a convenience sample of five pregnant women and five healthcare professionals; minor wording adjustments were made, but no items were added or removed.

The questionnaire was not formally validated, and no psychometric analyses (e.g., internal consistency, test–retest reliability, or construct validity) were performed.

Both questionnaires included identical items assessing knowledge of the fetal effects of four key micronutrients (folic acid, vitamin D, vitamin B12, and omega-3 fatty acids), as well as beliefs regarding whether adequate intake can be achieved through diet alone.

Additional items assessed supplementation practices among pregnant women and recommendation patterns among healthcare providers, as well as sources of information on pregnancy nutrition. The patient questionnaire further included demographic characteristics and dietary patterns, while the provider questionnaire collected data on professional background and clinical experience [20].

The primary outcomes were: (1) knowledge of micronutrient effects on fetal development, (2) beliefs regarding dietary sufficiency for key micronutrients, and (3) micronutrient supplementation practices (pregnant women) versus recommendation patterns (healthcare professionals). Secondary outcomes included sources of information on pregnancy nutrition and supplementation, as well as dietary habits.

### 2.3. Recruitment and Data Collection

QR codes linking to the respective questionnaires were displayed in antenatal clinic waiting areas, distributed during prenatal education sessions, and circulated via department-internal communication channels for healthcare professionals. The survey was self-administered and completed on personal smartphones or clinic devices. Participation was voluntary, and all responses were collected anonymously; no personal identifiers were collected. Before starting the questionnaire, respondents were informed that their anonymized data would be used for research purposes and provided electronic consent. The study was approved by the Ethics Committee of the University Hospital (Frankfurt Ethics Committee of the Johann Wolfgang Goethe-University Hospital Frankfurt number 2024-1732).

### 2.4. Participants

**Figure 1:**
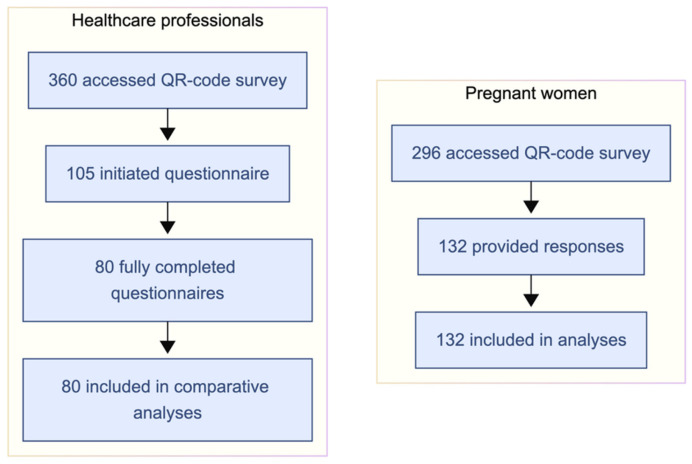
Participant Flow. Flow diagram of participant recruitment and inclusion in analyses. Comparative analyses of micronutrient knowledge, beliefs, and recommendation patterns were restricted to fully completed healthcare professional questionnaires.

Of 296 pregnant women who accessed the questionnaire via QR code, 132 provided responses and were included in the analyses (Figure 1). Of 360 healthcare professionals who accessed the survey, 105 initiated the questionnaire and 80 completed it fully (completion rate: 74.8%). Healthcare provider characteristics are reported for all respondents initiating the survey (*n* = 105), whereas comparative analyses of micronutrient knowledge, beliefs, and recommendation patterns were restricted to fully completed questionnaires (*n* = 80).

### 2.5. Statistical Analysis

Descriptive statistics are reported as absolute numbers and percentages (*n*, %). Group differences between pregnant women and healthcare professionals were assessed using the chi-square test or Fisher’s exact test, as appropriate. Healthcare provider characteristics were described for all respondents initiating the survey (*n* = 105), whereas comparative analyses of knowledge, beliefs, and recommendation patterns were restricted to fully completed questionnaires (*n* = 80). Not all questions were completed by every respondent; however, unless otherwise written, percentages are therefore calculated using the number of respondents who answered each item as the denominator, with item non-response reported as missing. Denominators vary due to item non-response. A two-sided *p*-value < 0.05 was considered statistically significant. Missing data were handled by complete-case analysis at the item level. No formal sensitivity analyses were conducted; therefore, the potential impact of non-random item non-response cannot be excluded. Due to the multiple-response format of the knowledge items, responses were not dichotomized into strictly “correct” or “incorrect.” Instead, responses were analyzed descriptively to identify patterns of knowledge, uncertainty, and misconceptions. Analyses were performed in GraphPad Prism 11 (GraphPad Software, San Diego, CA, USA).

## 3. Results

### 3.1. Participant Characteristics

A total of 132 pregnant women and 105 healthcare professionals initiated the survey. Comparative analyses of micronutrient knowledge, beliefs, and recommendation patterns were restricted to fully completed questionnaires, resulting in an analytical sample of 80 healthcare professionals.

The pregnant women were predominantly aged 26–35 years (88/132, 66.7%), reflecting the typical age of first-time mothers in the German university hospital setting (Table 1). The patient cohort was highly educated: 47.0% held a Master’s degree, 18.9% a PhD, and 18.9% a Bachelor’s degree, with only 2.3% reporting no formal educational qualification.

Diet type differed between groups, with most pregnant women (105/132, 79.5%) and just over half of healthcare professionals (58/105, 55.2%) following an omnivorous diet; 18.2% of pregnant women and 19.0% of providers reported a vegetarian or vegan diet. Almost all pregnant women (126/132, 95.5%) reported having actively sought information about nutrition during pregnancy, and 124/132 (93.9%) reported taking micronutrient supplements.

### 3.2. Patient–Provider Concordance in Micronutrient Knowledge

Across all micronutrients assessed, healthcare providers demonstrated higher levels of knowledge regarding fetal effects compared to pregnant women (Table 2). The most pronounced differences were observed for vitamin B12, vitamin D, and folic acid, whereas knowledge regarding omega-3 fatty acids did not differ significantly between groups. Provider knowledge was consistently higher than patient knowledge across all micronutrients, except for the misconception that folic acid influences bone development, which was more prevalent among patients (38.6% vs. 25.0%; *p* = 0.041).

Knowledge gaps were most pronounced for vitamin B12: over half of pregnant women (53.0%) reported not knowing any fetal effect of this micronutrient, compared to 20.0% of providers (Δ −33.0%; *p* < 0.001). Similarly, awareness that vitamin D is important for fetal bone development was substantially lower among patients than providers (54.5% vs. 80.0%; Δ +25.5%; *p* < 0.001), as was recognition of folic acid’s role in nervous system development (69.7% vs. 90.0%; Δ +20.3%; *p* ≤ 0.001).

For omega-3 fatty acids, a majority of both groups correctly identified a role in brain development (69.7% patients vs. 78.8% providers; Δ +9.1%; *p* = 0.150), and no statistically significant differences were observed across any response categories. However, uncertainty remained notable in both groups, with 25.0% of patients and 18.8% of providers selecting “don’t know”; 3.8% of providers but no patients selected “no influence” for omega-3 fatty acids.

### 3.3. Perceived Dietary Adequacy of Micronutrients

Both groups largely recognized that dietary intake alone is insufficient to meet requirements for folic acid and vitamin D during pregnancy, with no statistically significant differences between patients and providers for either micronutrient (folic acid: *p* = 0.452; vitamin D: *p* > 0.999) (Table 3). For omega-3 fatty acids, beliefs were also similar between groups (*p* = 0.128). Approximately half of patients and over half of providers considered dietary sources adequate, with a corresponding mirror pattern for those who judged diet alone to be insufficient.

In contrast, beliefs about vitamin B12 differed substantially and significantly between groups (*p* < 0.001). Pregnant women were approximately evenly split in their assessment of dietary sufficiency (61/129, 47.3% vs. 68/129, 52.7%), whereas the majority of healthcare providers (57/72, 79.2%) considered dietary intake alone adequate. This represents a marked discrepancy in the perceived need for vitamin B12 supplementation during pregnancy.

### 3.4. Patient-Provider Discordance in Micronutrient Supplementation Practices

A marked difference was observed between patient-reported supplementation practices and provider-reported recommendations. Across all four micronutrients, pregnant women reported substantially higher rates of supplementation than those recommended by healthcare providers (Table 4).

This discrepancy was most pronounced for vitamin B12, with 70.0% of pregnant women reporting supplementation, compared to only 3.8% of providers recommending it (Δ −66.2%; *p* < 0.001). Similar patterns were observed for vitamin D (76.2% vs. 41.3%; Δ −34.9 percentage points; *p* < 0.001) and omega-3 fatty acids, where supplementation rates among patients exceeded provider recommendations by more than 25 percentage points (76.2% vs. 47.5%; Δ −28.7 percentage points; *p* < 0.001). Folic acid represented the only micronutrient with high uptake in both groups, although patient-reported use (98.5%) still exceeded provider recommendation rates (81.3%, Δ −17.2 percentage points; *p* < 0.001).

### 3.5. Information Sources

**Figure 2:**
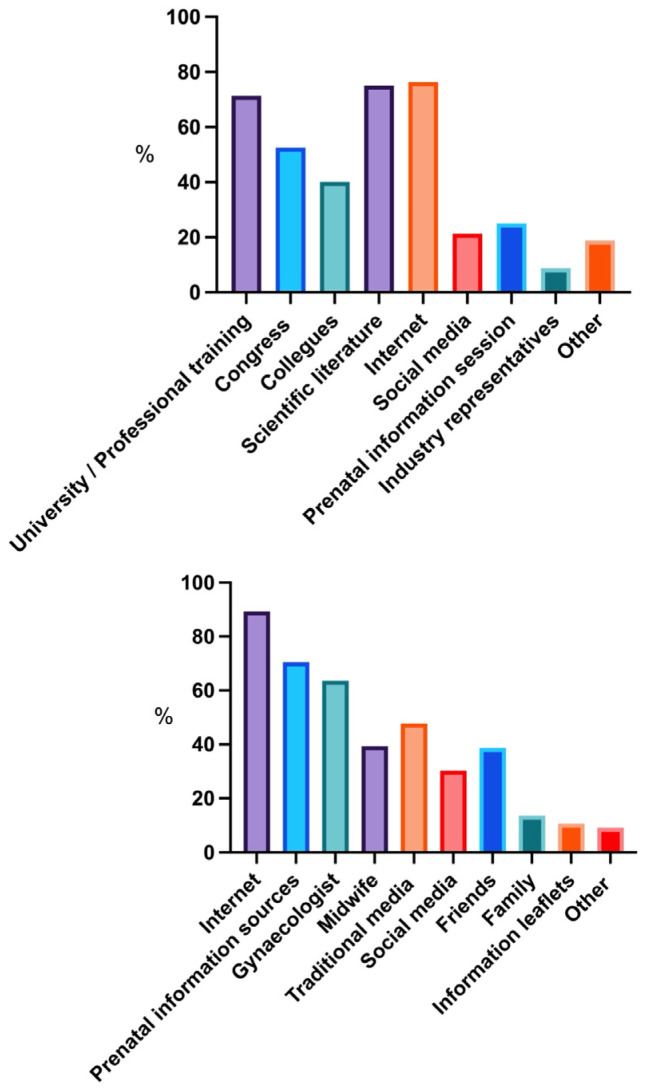
Information sources on nutrition in pregnancy reported by pregnant women (*n* = 132) and healthcare providers (*n* = 80). Multiple responses per participant were possible.

Nearly all pregnant women reported using multiple sources to obtain information on nutrition during pregnancy (Figure 2). The internet (89.4%) and prenatal information services (70.5%) were most frequently cited, followed by gynecologists (63.6%), midwives (39.4%), traditional media (47.7%), and social media (30.3%). Friends, family, information leaflets, and other sources were used less often. Among healthcare professionals, information is mainly derived from scientific literature (75.0%), the internet (76.3%), and university or professional training (71.3%), with congresses and colleagues as additional sources, while social media, prenatal information services, industry representatives, and other sources played a comparatively minor role.

## 4. Discussion

This study demonstrates substantial differences between pregnant women and healthcare providers across micronutrient-related knowledge, beliefs, and supplementation practices within a single tertiary care setting. Although healthcare professionals demonstrated higher overall knowledge levels across most assessed micronutrients, relevant gaps and uncertainties were observed in both groups, particularly regarding vitamin B12 and vitamin D.

Importantly, the present study assessed perceptions, beliefs, and self-reported practices rather than objective nutritional status or dietary intake. Therefore, the findings should be interpreted as reflecting differences in knowledge and perception rather than actual adequacy of micronutrient intake or adherence to evidence-based nutritional recommendations. In addition, the questionnaire was not formally validated, and no psychometric analyses were performed, which limits the interpretation of the measured knowledge constructs.

Although healthcare providers demonstrated higher overall knowledge levels, relevant gaps persisted in both groups, particularly regarding micronutrients beyond folic acid. The largest knowledge deficit was observed for vitamin B12 among pregnant women, with over half unable to identify any fetal effect; however, provider knowledge was also incomplete. These findings align with previous reports of variability in provider knowledge and counseling practices [13,21,22]. The high concordance observed exclusively for folic acid across knowledge, beliefs, and practices likely reflects the effectiveness of long-standing, consistent public health messaging, and the notably high supplementation rate in our cohort is consistent with previous reports from similarly educated populations [23,24,25].

A notable finding of the present study was the difference between patient-reported supplementation practices and provider-reported recommendation patterns. Across all four assessed micronutrients, pregnant women reported substantially higher supplementation rates than providers recommended, aligning with the literature [14,15,16,17]. The observed differences may reflect varying information sources and supplementation-related perceptions between pregnant women and healthcare professionals, potentially associated with widespread access to digital health information and over-the-counter supplement availability [14,15,22]. However, these measures do not represent directly equivalent constructs. Pregnant women reported personal supplementation behavior, whereas healthcare professionals reported general recommendation practices. Consequently, the observed differences should be interpreted as indicating potential misalignment rather than direct contradiction between patients and providers.

The internet was the most frequently cited information source among pregnant women (89.4%), underscoring the influence of non-clinical channels on supplementation decisions. Similar findings have been reported in other studies, where supplement use during pregnancy frequently exceeds clinical recommendations [25]. It should be noted that internet use among healthcare professionals likely reflects access to scientific literature, guidelines, and professional resources, which differ qualitatively from patient use of online information.

The findings for vitamin B12 warrant particular clinical attention. Despite limited knowledge of its fetal effects, 70.0% of pregnant women reported supplementation, while 79.2% of providers considered dietary intake alone adequate, and fewer than 4% routinely recommended it. This discrepancy likely reflects the prevailing assumption that vitamin B12 deficiency is relevant only in vegetarian or vegan women, an assumption reflected in current German guidelines. However, given that 18.2% of women in our cohort followed a vegetarian or vegan diet, and with plant-based diets becoming increasingly prevalent among women of childbearing age, the clinical relevance of vitamin B12 in routine prenatal care may be underestimated by healthcare professionals [5].

The absence of significant intergroup differences for omega-3 fatty acids suggests shared ambiguity in both groups, potentially reflecting the inconsistency of guideline recommendations for this micronutrient. In contrast, the high concordance observed for folic acid confirms that clear, long-standing, and widely disseminated public health recommendations are more reliably translated into both clinical recommendations and patient behavior [26].

From a clinical perspective, the observed dual pattern of under-guidance by providers and self-directed supplementation by patients raises concerns about both over- and under-supplementation. Patients may supplement without adequate guidance regarding indication, dosage, or clinical necessity, while providers may miss targeted counseling opportunities, particularly for vitamin B12 and vitamin D. Importantly, these discrepancies persisted even in a highly educated cohort, indicating that improved knowledge alone is unlikely to be sufficient. Behavior was not consistently aligned with either knowledge or provider recommendations, consistent with broader evidence that the relationship between knowledge and practice is mediated by multiple factors [22,25].

These findings suggest that micronutrient counseling during pregnancy may benefit from more structured and standardized communication approaches. Tools such as the FIGO Nutrition Checklist may represent one possible strategy to facilitate discussion of nutritional issues during antenatal care, although the present study did not directly evaluate the effectiveness of such interventions [27,28]. Embedding systematic micronutrient assessment into routine antenatal care may help ensure that key recommendations are addressed consistently and communicated effectively to pregnant women [22,27].

### Strength and Limitations

This study has several strengths. By assessing pregnant women and healthcare providers within the same institution using identical core knowledge items, it allows for a direct comparison that minimizes contextual variability. Recruiting from a highly educated patient population provides a stringent test of whether patient–provider alignment can be achieved under ostensibly favorable conditions.

Several limitations should be acknowledged. The single-center design and convenience sampling may limit generalizability. The response rate among pregnant women (44.6%) raises the possibility of selection bias, as individuals with a stronger interest in nutrition may have been more likely to participate. In addition, the study population was highly educated, which may limit generalizability to broader populations. All data were self-reported and may therefore be subject to reporting bias. Missing data were handled using complete-case analysis at the item level, and no formal sensitivity analyses were performed. While the patient questionnaire included items on information sources, including whether participants reported receiving nutritional information from a gynecologist or midwife, the study did not assess paired patient–provider interactions, the content or quality of individual counseling encounters, or whether discussions specifically addressed micronutrient supplementation. The study did not assess actual dietary intake or biomarker status. Consequently, no conclusions can be drawn regarding the effectiveness of nutritional counselling in the study population.

## 5. Conclusions

Differences in micronutrient-related knowledge, perceived dietary adequacy, and supplementation-related practices were observed between pregnant women and healthcare professionals within a tertiary care setting. Although healthcare professionals demonstrated overall higher knowledge levels, relevant uncertainties persisted in both groups, particularly regarding vitamin B12 and vitamin D. The findings further suggest potential differences between patient-reported supplementation behavior and provider-reported recommendation practices.

Given the exploratory and perception-based nature of the study, the findings should be interpreted cautiously. Nevertheless, they indicate that more structured communication approaches regarding micronutrient supplementation during pregnancy may be beneficial. Future studies incorporating dietary assessment, biomarker data, and longitudinal counseling evaluation are needed to better understand how micronutrient-related recommendations are translated into clinical practice and patient behavior.

## Figures and Tables

**Table 1:** Characteristics of study participants. Table 1 reports characteristics of all healthcare professionals initiating the survey (*n* = 105). Comparative analyses in subsequent tables were restricted to fully completed questionnaires (*n* = 80).

Characteristic	Pregnant Women (*n* = 132)	Healthcare Providers (*n* = 105)
*Age group*		
18–25 years	1 (0.8%)	1 (1.0%)
26–35 years	88 (66.7%)	44 (41.9%)
36–45 years	34 (25.8%)	40 (38.1%)
>45 years	2 (1.5%)	20 (19.0%)
Missing data	7 (5.3%)	0 (0.0%)
*Education*		
No degree	3 (2.3%)	—
University entrance qualification	7 (5.3%)	—
Bachelor’s degree	25 (18.9%)	—
Master’s degree	62 (47.0%)	—
PhD	25 (18.9%)	—
Missing data	10 (7.6%)	—
*Profession*		
Midwives	—	31 (29.5%)
OB/GYN specialists in hospital setting	—	32 (30.5%)
OB/GYN specialists in practice setting	—	17 (16.2%)
OB/GYN residents	—	23 (21.9%)
Medical students	—	2 (1.9%)
*Number of children*		
0	101 (76.5%)	—
1	22 (16.7%)	—
2 or more	4 (3.0%)	—
Missing data	5 (3.8%)	
*Diet type*		
Omnivore	105 (79.5%)	58 (55.2%)
Vegetarian/vegan	24(18.2%)	20 (19.0%)
Missing data	3 (2.3%)	27 (25.7%)
*Researched nutrition*		
Yes	126 (95.5%)	—
*Intake of micronutrients*		
Yes	124 (93.9%)	—

**Table 2:** Knowledge of fetal effects of micronutrients among pregnant women and healthcare providers. Analyses were restricted to fully completed healthcare professional questionnaires (*n* = 80). Δ = absolute difference in percentage points between pregnant women and healthcare providers. n/a = not applicable (no responses available). * *p* < 0.05, statistically significant.

Micronutrient/Fetal Effect	Patients *n* = 132 *n* (%)	Providers *n* = 80 *n* (%)	Δ (%)	*p*-Value
*Omega-3 fatty acids*				
Brain development	92 (69.7%)	63 (78.8%)	+9.1	0.150
Bone development	11 (8.3%)	9 (11.3%)	+3.0	0.481
Nervous system development	53 (40.2%)	39 (48.8%)	+8.6	0.254
No influence	0 (0.0%)	3 (3.8%)	+3.8	0.053
Don’t know	33 (25.0%)	15 (18.8%)	−6.2	0.315
*Folic acid*				
Brain development	43 (32.6%)	41 (51.3%)	+18.7	0.009 *
Bone development	51 (38.6%)	20 (25.0%)	−13.6	0.041 *
Nervous system development	92 (69.7%)	72 (90.0%)	+20.3	<0.001 *
No influence	0 (0.0%)	0 (0.0%)	0.0	n/a
Don’t know	16 (12.1%)	3 (3.8%)	−8.3	0.047 *
*Vitamin D*				
Brain development	22 (16.6%)	19 (23.8%)	+7.2	0.206
Bone development	72 (54.5%)	64 (80.0%)	+25.5	<0.001 *
Nervous system development	22 (16.7%)	20 (25.0%)	+8.3	0.140
No influence	1 (0.8%)	2 (2.5%)	+1.7	0.558
Don’t know	44 (33.3%)	15 (18.8%)	−14.5	0.027 *
*Vitamin B12*				
Brain development	32 (24.2%)	37 (46.3%)	+22.1	0.001 *
Bone development	21 (15.9%)	16 (20.0%)	+4.1	0.460
Nervous system development	35 (26.5%)	48 (60.0%)	+33.5	<0.001 *
No influence	2 (1.5%)	2 (2.5%)	+1.0	0.634
Don’t know	70 (53.0%)	16 (20.0%)	−33.0	<0.001 *

**Table 3:** Perceived adequacy of dietary intake during pregnancy.

Micronutrient	Patients n/N (%)	Providers n/N (%)	Δ (%)	*p*-Value
*Folic acid*				
Yes, sufficient	27/128 (21.1%)	11/71 (15.5%)	−5.6	0.452
No, not sufficient	101/128 (78.9%)	60/71 (84.5%)	+5.6	
*Vitamin D*				
Yes, sufficient	17/129 (13.2%)	9/75 (12.0%)	−1.2	>0.999
No, not sufficient	112/129 (86.8%)	66/75 (88.0%)	+1.2	
*Omega-3*				
Yes, sufficient	61/129 (47.3%)	38/64 (59.4%)	+12.1	0.128
No, not sufficient	68/129 (52.7%)	26/64 (40.6%)	−12.1	
*Vitamin B12*				
Yes, sufficient	61/129 (47.3%)	57/72 (79.2%)	+31.9	<0.001 *
No, not sufficient	68/129 (52.7%)	15/72 (20.8%)	−31.9	

Values are n/N (%). Δ indicates the absolute difference in percentage points. Denominators vary due to item non-response. Analyses were restricted to fully completed healthcare professional questionnaires (*n* = 80). * *p* < 0.05, statistically significant.

**Table 4:** Comparison of supplementation practices (pregnant women) and recommendations (healthcare providers).

Micronutrient	Patients n/N (%)	Providers n/N (%)	Δ (%)	*p*-Value
Folic acid	128/130 (98.5%)	65/80 (81.3%)	−17.2	<0.001 *
Vitamin D	99/130 (76.2%)	33/80 (41.3%)	−34.9	<0.001 *
Omega-3	99/130 (76.2%)	38/80 (47.5%)	−28.7	<0.001 *
Vitamin B12	91/130 (70.0%)	3/80 (3.8%)	−66.2	<0.001 *

Values are n/N (%). Δ indicates the absolute difference in percentage points. Denominators vary due to item non-response. Analyses were restricted to fully completed healthcare professional questionnaires (*n* = 80). * *p* < 0.05, statistically significant.

## Data Availability

The data are available from the corresponding author upon reasonable request.
